# Self-Medication Practice and Associated Factors among Residents in Wuhan, China

**DOI:** 10.3390/ijerph15010068

**Published:** 2018-01-04

**Authors:** Xiaosheng Lei, Heng Jiang, Chaojie Liu, Adamm Ferrier, Janette Mugavin

**Affiliations:** 1 School of Management, Hubei University of Chinese Medicine, Wuhan 430065, China; 2 Centre for Alcohol Policy Research, School of Psychology and Public Health, La Trobe University, Melbourne, VIC 3086, Australia; J.Mugavin@latrobe.edu.au; 3 Melbourne School of Population and Global Health, University of Melbourne, Melbourne, VIC 3010, Australia; 4 Department of Public Health, School of Psychology and Public Health, La Trobe University, Melbourne, VIC 3086, Australia; C.Liu@latrobe.edu.au (C.L.); adamm.ferrier@latrobe.edu.au (A.F.)

**Keywords:** self-medication, practice, associated factors, Chinese residents

## Abstract

Background: This study aims to examine the prevalence and predictors associated with self-medication, and related consequences in Wuhan, China. Methods: Two-hundred-sixty residents were interviewed from randomly selected four districts of Wuhan, China. A modified version of Anderson’s health behavioral model was used in the survey to collect information of self-medication behavior. Multivariable logistic regression analyses were used to measure correlates of the prevalence of self-medication. Results: Nearly half of the respondents would select self-medication, and 39.1% would see a doctor if they felt sick. The most common self-medicated illnesses were cold and cough, cardiovascular disease and gastrointestinal disease. The main reasons for self-medication were that the illness was not severe (enough) to see the doctor (45%); the patient did not think that the trouble of seeing a doctor was worth the effort (23%); the patient had no time to see the doctor (12%), and the patient did not want to pay high medical costs (15%). Logistic regression results suggested that respondents tended to select self-medication if the illness was minor or short-term (less than seven days). Conclusions: Our findings suggest that more strict regulation on over-the-counter medicines may be required to reduce health risks related to self-medication. Targeted health education on the risks of self-medication should be considered.

## 1. Introduction

Self-medication plays an important role in health care. It has a positive impact on individual health and the health care system [1]. For minor illnesses, it often provides a cheap, rapid, and convenient solution, without which the health care system of any country would be overcome with demand. Self-medication could save waiting times for consumers to see the doctor, and save scarce medical resources from being used on minor conditions [2]. However, irrational self-medication practice may increase health risks such as misdiagnosis, drug resistance and interactions, delays in seeking medical advice, adverse drug reactions, and polypharmacy [3,4,5,6].

Self-medication has been increasingly popular in China in the last two decades due to (1) increases in the costs of professional medical services, (2) an aging population and an over-complicated health service delivery in China, (3) overtreatment, damaging the credibility of health institutes, (4) a lack of health insurance coverage and a poor quality of health care system at the community level [7,8,9]. The 2008 China National Health Services Survey reported that 38% of people who were ill for a period of two weeks did not seek medical assistance, and of these, 70% opted for self-medication [10]. The Chinese Nonprescription Medicines Association reported in 2011 that improper medication practices existed in over 70% of Chinese families, and the rate of irrational drug use ranged from 12 to 32% [11].

Self-medication occurs throughout the world. A survey in Britain reported that 93% of patients experienced body pain within one month, and of these 75% self-prescribed an over-the-counter (OTC) analgesic [12]; 72% of patients with a cold, cough, and headache in the United States would choose to self-medicate in the first instance [13]. The prevalence of self-medication was 75% in Chile [14], 65% in Brazil [1], and 53% in Mexico [15]. The major risks caused by irrational self-medication include misdiagnosis and incorrect drug selection, non-compliance with guidelines, and hoarding drugs. Previous studies found that many people relied on their own previous experiences or their choices based on the recommendations of friends, some believing that “experience makes the patient the best doctor” [16]. If the patient’s diagnosis of their condition is incorrect, their consequential drug selection may aggravate their condition, or even cause additional problems that may make subsequent medical intervention problematic. Whilst understanding the pharmaceutical properties of different medications is a critical basis for appropriate drug selection according to need, compliance with administration guidelines is often lacking. People may randomly prolong use, misuse OTC drugs, or combine treatment with other contraindicated drugs, leading to adverse interactions and reactions. Hoarding of drugs is also problematic. Unused medications are often kept well beyond their use-by dates, or stored without appropriate identification (such as mixed together in a container without the original packaging) which can contribute to potential misuse. Furthermore, 21% of drugs stored in family medical kits have often expired, and drug packaging insert was missing (18%) [17]. Therefore, irrational self-medication has become an important public health problem in many countries, as well as in China [18], and its health risk should arouse wide attention of the society.

The prevalence of self-medication was found to be negatively associated with private health insurance coverage in Mexico [19]. Decision to opt for self-medication is influenced by many factors, such as education level, family and society influence, availability of drugs, and exposure to advertisements [20]. People’s experience of severity and duration of illness may also influence the opting of self-medication [21]. Although the prevalence and correlates of self-medication have been widely discussed in many countries [22,23,24], the risk and adverse effects of irrational self-medication, and the key influencing factors that can affect opting for self-medication among Chinese residents, have been rarely discussed.

The current study aims to assess self-medication behavior and its associated factors among Chinese residents in Wuhan, China. The findings of the study will provide research evidence to inform health policy and medication practice to reduce health risks related to self-medication.

## 2. Methods

The Wuhan Self-Medication (SM) Survey was conducted during September and October 2015 in Wuhan city, which is the most populous city in Central China with a population of over 10 million in 2015. A cluster sampling method was used. The required sample size, calculated based on the effect size of 0.2 with 95% statistical power, is 260 participants. We randomly selected 4 out of the 13 districts in Wuhan to conduct investigation using the lottery method. Two hundred and sixty participants (65 in each district) were interviewed face to face after investigators obtained verbal consent from participants. The inclusion criteria for study participants were adults (aged 18+) living in the surveyed community with basic reading and comprehension skills. In total, 258 interviews were completed (a completion rate of 99.2%). Survey questionnaires with incomplete information were excluded. In this study, self-medication was defined as people using at least one OTC drug (including Chinese and non-Chinese medicines, but excluding Chinese supplements/food products being used for therapeutic purposes) to treat self-recognized illnesses or symptoms.

### 2.1. Study Framework

**Figure 1:**
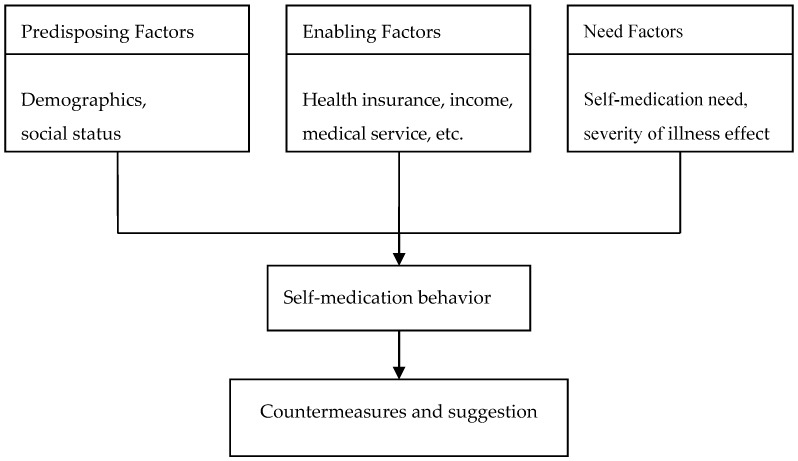
Conceptual framework of the modified Andersen model of health service utilization.

The questionnaire was based on the modified Andersen model of health service utilization [25]. This model assessed people’s option to use health services depending on three factors (see Figure 1): predisposition, enabling, and needs. Predisposing factors include sex, age, ethnicity, socioeconomic status, occupation, and education. Enabling factors refer to availability constraints such as household income, medical insurance, medical services, and proximity to medical services. Need factors are those which trigger the action to self-medicate, such as type of illness and duration of illness.

### 2.2. Measures and Statistical Analysis

In our analysis, the dependent variable is the prevalence of self-medication (if you had a symptom or health condition during the two-week period preceding the survey, did you chose to (1) self-medicate, (2) see the doctor, or (3) do nothing). Independent variables include age, gender, occupation, marital status, education, income, level of medical insurance, distance to medical services, severity of disease, and length of illness. Age was divided into four groups. Education was measured as the highest degree that the respondents had completed. Monthly income was expressed in Chinese currency. Medical insurance was measured by different levels of insurance that respondents had in China. Distance to medical unit was measured as the kilometers from residence to the nearest medical services. Severity of disease was measured by self-perception of disease status (1 = severe; 2 = common; 3 = mild). Length of illness was measured as the number of sickness days. The study variables and their measure codes are described in Table S1 in Supplementary Materials.

Data were analyzed using Statistical Package for Social Sciences (SPSS) version 19.0. Descriptive statistics were used to describe respondent’s self-medication behavior. Chi-square test, correlation analysis, and multivariable logistic regression analysis were conducted to identify influencing factors of self-medication.

## 3. Results

### 3.1. The Basic Statistics of Study Participants

The descriptive statistics of study sample are shown in Table 1. Of 258 respondents, 49.2% were male and 50.8% were female. The majority of respondents were aged between 21 and 60 (21–40 (36.4%); 41–60 (42.6%)). The occupation of respondents included enterprise staff and small business owners (36%) as well as workers and peasants (24%). Most of the respondents (72.1%) were married. More than half (57%) of respondents had graduated from college or university or above, while 21.3% had only a high school or secondary school education. Thirty-three percent of the respondents earned a monthly income of 3001–4500 Chinese Yuan, while 26.7% reported a monthly income of 1500–3000 Chinese Yuan. The majority (97.3%) of the respondents had some type of medical insurance such as urban basic medical insurance (69%), a new cooperative medical scheme (NCMS) (18.2%), and commercial medical insurance (4.3%). Few respondents had no health insurance (2.7%).

### 3.2. Self-Medication Practice

**Figure 2:**
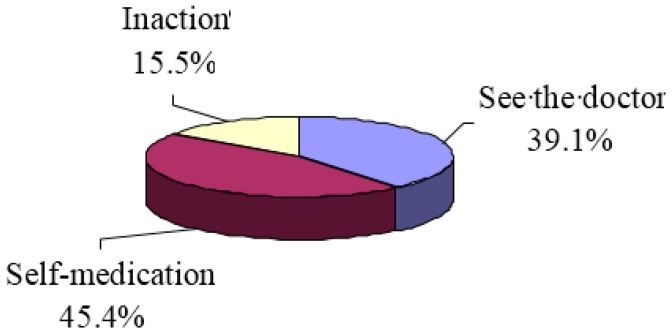
Respondent’s medical treatment choice.

**Figure 3:**
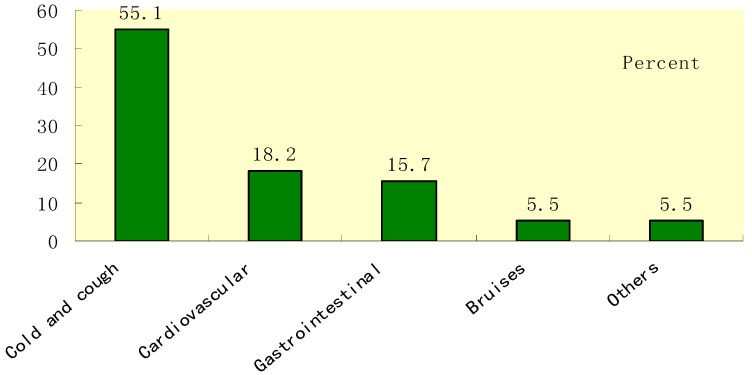
Type of illness for self-medication.

Among respondents who were asked if they felt physical discomfort in the 2 weeks preceding the survey, 39.1% of the respondents would see the doctor, 45.4% would select self-medication, and 15.5% would select inaction or do nothing (shown in Figure 2). In the previous three years, almost all of the respondents had the experience of self-medication, among which 230 (89%) mainly treated minor illnesses and chronic diseases. Figure 3 shows the major ailments reported for self-medication: cold and cough (55.1%), cardiovascular and cerebrovascular diseases (18.2%), gastrointestinal diseases (15.7%), and bruises (5.5%).

The main reasons of opting for self-medication reported by the respondents include the following: that the severity of the illness did not warrant a visit to the doctor (45.4%); that the trouble of seeing a doctor was not worth the effort (22.5%); that there was no time to see a doctor (11.6%); that medical costs were too high (15.1%) (Table 2). The sources of drug information for self-medication were from past experience (51.2%), a friend’s recommendation/peer advice (27.7%), the Internet (19.1%), and newspapers and magazines (2%). According to the survey, the respondent’s household mean cost for self-medication was 1017.6 Chinese Yuan in the last 12 months, accounting for 36.5% of the average household annual medical expense. The average frequency of household purchasing drugs from pharmacy was 2.1 times in the previous 12 months. About 94.5% of the respondents believed self-medication was effective most of the time, whereas 1.2% thought it was ineffective, and 4.3% were uncertain about it.

### 3.3. Adverse Drug Reaction of Self-Medication

Among the respondents, 79.5% had read the instructions (i.e., the ‘directions for use of the over-the-counter medication’), while 20.5% had not read the instructions. Eighteen percent of respondents who self-medicated reported a self-perceived adverse drug reaction (Table 3). Respondents who read the instructions were less likely to report an adverse drug reaction than those who did not read the instructions (*X*^2^ = 4.99, *p* = 0.03). There was a significant association between whether or not instructions were read and education level (*X*^2^ = 45.83, *p* = 0.00), with results suggesting that higher educated respondents were more likely to read the instructions of the drug (Table 4).

### 3.4. Regression Analysis on the Influencing Factors of Self-Medication

Multivariable logistic regression analyses were used to identify which factors can affect opting for self-medication. The results showed that the respondents with the more serious or severe diseases and the longer duration of illness were less likely to choose self-medication (*p* < 0.05). For short-term or minor illnesses, respondents tended to select self-medication, whereas when length of ailments was more than 7 days or diseases became severe, the cases of self-medication were reduced (Table 5). Variables such as age, gender, occupation, marital status, education level, income, medical insurance, and accessibility of medical institutions had no correlation with opting to self-medication.

## 4. Discussion

Self-medication is a common behavior among residents of Wuhan in China. The most common illnesses/reasons for self-medication were minor and chronic diseases such as a cold or cough, gastrointestinal diseases, and cardiovascular and cerebrovascular diseases. The study revealed that the prevalence of self-medication, with a recall period of two weeks, was 45.4% in Wuhan, which was higher than that of seeing the doctor (39.1%). Similar results have been reported in other studies, e.g., 75% in Chile [14], 65.1% in Brazil [1], 53.5% in Mexico [15], 79.9% among university students in Serbia [26], and 42.5% in Jordan [27]. This study showed that self-medication was mainly used to treat common ailments and chronic diseases, such as a cold, cough, gastrointestinal diseases, and cardiovascular and cerebrovascular diseases, which was consistent with previous studies [28,29]. For patients who have previously had a heart attack or a stroke or other cardiovascular and cerebrovascular diseases, rational self-care and self-medication was found to help to prevent future cardiovascular events and strokes [30].

When a disease was more severe, self-medication cases were substantially reduced. The reasons for choosing self-medication were as follows: that the disease was not severe enough to visit a doctor, that the trouble of seeing a doctor was not worth the effort, that there was no time to go to the medical or health clinic, and that medical costs were too high. The majority of respondents performed self-medication because they felt the disease was too mild to require medical service. This finding is frequently reported by other studies [27,29]. Some respondent’s work schedule matched the clinic’s opening hours, so attending a medical appointment (during work hours) was problematic [31]. For respondents without medical insurance or with a lower level of medical insurance cover, self-medication could reduce or minimize costly medical care.

Irrational self-medication is related to a certain health risk. Though the majority of respondents (94.5%) believed that self-medication was effective, 46 (17.8%) experienced an adverse drug reaction (ADR) in self-medication. Furthermore, ADR was significantly related to whether or not the respondent had read the drug instructions (*p* < 0.01). Respondents who did not read the drug instructions were more likely to report an ADR. There was a significant difference in whether or not respondents read the instructions and education levels (*p* < 0.01). Respondents with a lower level of education, compared with those with a high level of education, were significantly less likely to read the instructions. The results for sources of self-medication knowledge suggest that the majority of respondents (51.2%) relied on their past experiences, which is consistent with a previous investigation [28]. Others gathered information from a friend’s recommendation/peer advice, the Internet, or newspapers and magazines. The same or similar disease symptoms may be caused by various etiologies, so relying on past experience might lead to misdiagnosis and ADR.

Self-medication was associated with various factors. Findings from multivariable regression suggest that the severity of the illness and the duration of the disease were significantly correlated with the prevalence of self-medication (*p* < 0.01). This finding is in line with a previous study on self-treatment in China [7]. For short-term or minor illnesses, respondents tended to select self-medication. When the duration of the illness was more than 7 days, the use of self-medication was reduced. Furthermore, when disease severity increased, respondents were less likely to choose self-medication.

Previous studies claimed that insurance is a key determinant for self-treatment [7,32]. However, this correlation was not found in self-medication analysis, a possible reason is that most OTC drugs (90%) used for self-medication in China were not covered by medical insurance. For example, the three major medical insurance sources in Chinese cities (NCMS, urban basic medical insurance, and free medical insurance) mainly cover residents’ outpatient and inpatient costs. Broadening health insurance coverage on the number of OTC drugs may lead to a significant impact on self-medication behavior and may result in cost and resource reductions for the health and medical system.

### Limitations

The recall bias may have affected our analysis. However, survey interviewers were trained to use either a local dialect or Mandarin in the interview to minimize information bias. A number of factors with the potential to affect respondents’ opting for self-medication were not included in the study questionnaire, including the quality of health services (perceived quality of care) and the influence of family and their health behavior (chronic illness, smoking, drinking, and physical activity). Furthermore, the small sample size in this study led to wide 95% confidence intervals in our analysis. Thus, the study results should be treated with caution. This study is only a preliminary characterization of self-medication in Wuhan, China. The next step is to focus on self-medication among priority groups, such as the elderly, women, and children, and those with chronic diseases, and such studies should be based on a large, representative sample size.

## 5. Conclusions

This study has revealed that over 45% of the respondents would select self-medication if they felt sick in Wuhan, China. Cold and cough, cardiovascular disease, and gastrointestinal disease were the most common illnesses suffered by respondents opting for self-medication, and respondents tended to select self-medication if the illness is minor or short-term (less than seven days). The research findings suggest that regulations on drug instructions should be strengthened by the department of public health. The pharmacist in his or her professional capacity is competent enough to provide customers sound advice on the medicine being supplied [33]. A standardized drug consultation service system is needed, providing consumers with professional drug consulting services so as to reduce the health risk of self-medication. Further actions are needed to prevent harms of irrational self-medication in China, such as improving the accessibility of medical services and the coverage of health insurance, developing community health services, and controlling the increasing medical expenses.

## Figures and Tables

**Table 1:** Characteristics of the study respondents (N = 258).

Characteristics	Frequency	Percentage
Gender		
Male	127	49.2
Female	131	50.8
Age (years)		
18–20	17	6.6
21–40	94	36.4
41–60	110	42.6
>60	37	14.4
Occupation		
Worker	40	15.5
Peasant	22	8.5
Teacher	29	11.2
Civil servant	19	7.4
Medical staff	8	3.1
Business owner	33	12.8
Enterprise staff	60	23.3
Others	47	18.2
Marital Status		
Married	186	72.1
Unmarried	72	27.9
Education		
Middle school and lower	56	21.7
High/Secondary School	55	21.3
College/University	131	50.8
Master and above	16	6.2
Monthly income (Chinese Yuan ¥)		
<1500	19	7.4
1500–3000	69	26.7
3001–4500	86	33.3
4501–6000	56	21.7
>6000	28	10.9
Medical insurance		
Urban basic medical insurance	178	69.0
Free medical insurance	15	5.8
New cooperative medical scheme	47	18.2
Commercial medical insurance	11	4.3
No medical insurance	7	2.7

**Table 2:** The reason and source of knowledge for self-medication.

Variables	Frequency	Percentage
The reason of self-medication *		
High medical costs	39	15.1
Save the trouble of seeing a doctor	58	22.5
Mild disease	117	45.4
No time to see the doctor	30	11.6
Other reasons	14	5.4
*Subtotal*	*258*	*100.0*
Medication knowledge sources *		
Advice from others or peers	71	27.7
Internet	49	19.1
Newspaper and magazine	5	2.0
Past experience	131	51.2
*Subtotal ^#^*	*256*	*100.0*

Notes: * *p* < 0.05 and a chi-square test were conducted to test categorical difference. ^#^ The sample size is 256 because of 2 missing values.

**Table 3:** Adverse drug reaction and whether the instructions were read or not (person (%)).

Variable	Read Instruction	Not Read Instruction	Total
Adverse drug reaction *			
Yes	31 (15.1)	15 (28.3)	46 (17.8)
No	174 (84.9)	38 (71.7)	212 (82.2)
Total	205 (100)	53 (100)	258 (100)

Note: * *p* < 0.05 and a chi-square (*X*^2^) test were conducted to test categorical difference.

**Table 4:** The situation of reading instructions in different education level (person (%)).

Variable	Read Instruction	Not Read Instruction	Total
Education level *			
Junior high school or below	27 (48.2)	29 (51.8)	56 (100)
High school, secondary school	44 (80.0)	11 (20.0)	55 (100)
Training certificate, bachelor or higher	134 (91.2)	13 (8.8)	147 (100)
Total	205 (79.5)	53 (20.5)	258 (100)

Note: * *p* < 0.05 and a chi-square test were conducted to test categorical difference.

**Table 5:** Multivariable logistic regression analyses on influencing factors of self-medication.

Independent Variable (N = 258)	*β*	*SE*	Wald *X*^2^	*p*	OR	(95% CI)
Age	−0.14	0.22	0.44	0.51	0.87	(0.57, 1.32)
Gender (male/female)	−0.21	0.27	0.63	0.43	0.81	(0.48, 1.37)
Employment (employed/unemployed)	−0.03	0.06	0.32	0.58	0.97	(0.86, 1.09)
Marital status (yes/no)	−0.26	0.13	4.24	0.04	0.78	(0.60, 0.99)
Education (high school or lower/college, bachelor or higher)	0.32	0.18	3.04	0.08	1.37	(0.96, 1.96)
Income (≤6000/>6000)	0.04	0.13	0.10	0.75	1.04	(0.81, 1.34)
Medical Insurance (yes/no)	−0.50	0.81	0.37	0.54	0.61	(0.12, 3.02)
Distance to medical units (<4 miles/≥4 miles)	−0.15	0.11	1.96	−0.15	0.86	(0.69, 1.06)
Severity of disease (severe, common/mild)	−0.63	0.24	6.65	0.01	0.53 **	(0.33, 0.86)
Length of illness (<7 days/≥7 days)	0.46	0.16	8.78	0.00	1.59 **	(1.17, 2.16)
Constant	1.89	1.65	1.32	0.25	6.65	

Notes: SE is standard error; Log likelihood = 355.428; Log likelihood *X*^2^ = 26.504; Cox & Snell pseudo R^2^ = 0.098; ** *p* < 0.01.

## Supplementary Materials

The following are available online at www.mdpi.com/1660-4601/15/1/68/s1, Table S1: Variable names and measures.
