# Supplementary material for: Self-Medication Practice and Associated Factors among Residents in Wuhan, China
Source: Int J Environ Res Public Health. 2018 Jan 4;15(1):68. doi: 10.3390/ijerph15010068 (PMC5800167; doi:10.3390/ijerph15010068)
Supplement: Supplementary file 1 [file ijerph-15-00068-s001.pdf]

Table S1. Variable names and measures

Variable name	Measure
**Dependent variable :** Self-medication	1=Self-medication; 2=Non self-medication
**Independent variable:**	
Age	1="18-20"; 2="21-40"; 3="41-60"; 4=">60"
Gender	1 = Male; 2 = Female
Occupation	1 = Worker; 2 = Peasant; 3 = Teacher; 4 = Civil servants; 5 = Medical staff; 6= small business owner; 7= Enterprise staff; 8= Unemployed
Marital status	1 = Married; 2 = Unmarried
Education	1 = Middle school and lower; 2 = High/Secondary School; 3 = College/University; 4 = Master and above;
Monthly income (Chinese Yuan)	1 = "<1500"; 2 = "1500-3000"; 3 = "3001-4500" 4 = "4501-6000"; 5 = ">6000"
Medical insurance	1 = Urban basic medical insurance; 2 = Free medical care; 3 = New cooperative medical scheme (NCMS); 4 = Commercial insurance; 5 = Uninsured
Distance to medical unit （km）	1 = "＜1"; 2 = "＜2"; 3 = "＜3"; 4 = "＜4"; 5 = "≥4"
Severity of disease	1 = severe; 2 = common; 3 = mild
Length of illness (days)	1 = "＜3"; 2 = "＜5"; 3 = "＜7"; 4 = "≥7"
